# A peanut quality control material to improve allergen analysis – How difficult can it be?

**DOI:** 10.1186/2045-7022-5-S3-P116

**Published:** 2015-03-30

**Authors:** Michael Walker, Gill Holcombe, Deborah House, Joanna Topping, Clare Mills

**Affiliations:** 1 LGC, Belfast, United Kingdom; 2 Manchester University, Manchester, United Kingdom

## 

Flawed allergen analysis hinders detection and measurement of allergens, which is important for allergen risk management in food labelling, in-factory cleaning and threshold levels. ELISA, DNA and MS can be used to detect food allergens but there are quantification problems with all three. ELISA is currently the most commonly applied technique however, although it can detect the presence of most protein allergens in foods, it can struggle with recovery and accurate quantification. A well characterised quality control material, ideally a certified reference material, has been called for by many to improve consistency in analytical results for allergens. However there are practical difficulties with production which are often overlooked, including ensuring sufficient homogeneity and long term stability, assessing the incurred quantity, and maintaining a relationship with the concentrations that affect allergy sufferers. The authors present a successful study which is a first step towards addressing the above problems. A prototype quality control set, (a blank material and a QC material with peanut protein added at 10 mg kg^-1^ level) has been prepared based on a EuroPrevall study matrix used to assess clinical thresholds[1]. The homogeneity and stability of the material have been evaluated and assessed as satisfactory and the material is now available to the wider analytical community. This is valuable progress in addressing the lack of allergen related incurred reference materials to improve global allergen protein measurement.
